# Spiritual Care at the Crossroads: An Ecumenical White Paper on the Future of Christian Healthcare Chaplaincy

**DOI:** 10.1007/s10943-025-02255-0

**Published:** 2025-02-25

**Authors:** Simon Peng-Keller, Michael Balboni, Tracy Balboni, Annette Haussmann, Trace Haythorn, Pascal Mösli, David Neuhold, Daniel R. Nuzum, Wim Smeets, Chris Swift, John Swinton, Traugott Roser, Anne Vandenhoeck, Fabian Winiger

**Affiliations:** 1 Faculty of Theology and the Study of Religion, University of Zurich, Kirchgasse 9, 8001 Zurich, Switzerland; 2 Harvard Medical School Boston, Boston, MA USA; 3 Faculty of Theology, Heidelberg University, Heidelberg, Germany; 4 Chaplaincy Innovation Lab, Decatur, GA USA; 5 Faculty of Theology, University of Lucerne, Lucerne, Switzerland; 6 Department of Obstetrics & Gynaecology, University College Cork College of Medicine and Health, Cork, Ireland; 7 Clinical Pastoral Education, Cork University Hospitals Group, Cork, Ireland; 8 Radboud University, Nijmegen, NL the Netherlands; 9 Staffordshire University, York, UK; 10 School of Divinity, History and Philosophy, King’s College, University of Aberdeen, Aberdeen, UK; 11 Faculty of Protestant Theology, University of Münster, Münster, Germany; 12 Faculty of Theology and Religious Studies, KU Leuven, Louvain, Belgium

**Keywords:** Specialized spiritual care, Professionalization, Spiritual diversification, Spiritual care competencies, Chaplaincy education and training

## Abstract

This ecumenical White Paper aims to clarify the profile of Christian healthcare chaplaincy in a context of rapidly changing healthcare systems. The increasing complexity and specialization of healthcare, the shift towards outpatient care, mounting economic pressures, the demographic and epidemiological transitions towards chronic and elderly care, and global health crises are profoundly transforming and challenging Christian healthcare chaplaincy. At the same time, the societal context of healthcare chaplaincy is also changing rapidly. While church resources and influence are declining, spiritual and religious diversification is on the rise. This paper engages with this rapid change as a moment of opportunity. It addresses the role of Christian healthcare chaplains in a time of change and clarifies their theological basis, their professional competencies, and the future of their education and training.

## Introduction: Goals and Background of this White Paper

This White Paper aims to stimulate critical discussion among all those committed to renewing healthcare chaplaincy in a time of change. The increasing complexity and specialization of healthcare, the shift towards outpatient care, mounting economic pressures, demographic and epidemiological transitions towards chronic and elderly care, and global health crises such as the COVID-19 pandemic are transforming and challenging healthcare chaplaincy in many ways.

At the same time, the societal context of healthcare chaplaincy is also changing rapidly. Church resources and influence are declining in many parts of the world. De-institutionalization, de-traditionalization, and secularization will continue to challenge faith communities profoundly. Simultaneously, we are witnessing an ongoing diversification of religious, spiritual, and secular life, both within and outside faith communities, and the emergence of an understanding of the “spiritual” that transcends the traditional distinction between religious and secular spheres. Chaplains are challenged to engage with a growing diversity of spiritualities in their work and to practice “code-switching” while at the same time situating themselves in a changing religious-spiritual field.

Healthcare chaplains are located at the intersection of these two momentous transformation processes according to their respective national, regional, and institutional contexts. This paper is intended to engage with this rapid change as a moment of opportunity. The key challenges discussed on the following pages are common to healthcare chaplains with diverse backgrounds: first, to navigate religious and spiritual diversity compassionately and in a way that is sensitive to individual needs and cultural and personal differences, and secondly, to do so from their rootedness in particular faith traditions and spiritual practices.

The authors of this White Paper welcome all efforts that strengthen the professionalism and diversity of healthcare chaplaincy. Due to our backgrounds and expertise, we are focusing on one particular group: healthcare chaplains who are anchored in and inspired by Christian spirituality in its different variants. While this White Paper is written primarily with *this* group of chaplains in mind, several of its central points are relevant for spiritual care providers in other care settings and rooted in other faith traditions. As such, it also contributes to developing the broader spiritual care discourse. We adopt the view of the *World Council of Churches* that the “Christian ministry of healing belongs primarily to the whole fellowship of the church, and only in that context to those who are specially trained for it” (World Council of Churches, 1965, p. 35). Thus, we understand Christian healthcare chaplains^1^ as specialized professionals with a double belonging: On the one hand, they belong to an inter- and trans-religious professional group in the healthcare sector, and, on the other hand, to the Christian faith community that inspires and, in many cases, mandates their work. This White Paper responds to the tension that arises from this dual belonging and argues that it should be consciously and creatively embraced. The “Christian call to healing” (Mark 6:7–12; Matthew 10:1, 7–11; Luke 9:1–6; 10:1–12) refers to the whole spectrum of caring for the sick, including reducing their marginalization. It encompasses all forms of support for sick, disabled, dying, and marginalized people regardless of their faith and religious affiliation. It is inspired by the experience of being reconnected, comforted, strengthened, and healed in a comprehensive manner.

But how can this call be realized in a healthcare system dominated by biomedicine and struggling with the aforementioned societal changes? One can argue with the Book of Jesus Sirach 38:1–15 that the Christian contribution consists in a particular motivation and approach, and not in specific methods: God heals and cures also by means of modern medicine, nursing, psychology, physiotherapy, and any other appropriate methods available to us. Understanding and practicing one’s health profession as a vocation thus does not mean distrusting evidence-based procedures, but rather, being guided by a Christian ethos, and using them to the best of our knowledge and ability to the benefit of all. Healthcare chaplaincy can fulfil both its divine calling and professional mandate when it is prepared to avail itself of modern psychological knowledge and counselling skills alongside traditional spiritual resources.

In the following, we aim to take Christian healthcare chaplaincy a step further, highlighting the life-changing character of the presence of God’s spirit. The Christian call to healing, which can be answered by different actors (chaplains, health professionals, volunteers, and not least, patients and family members), is met in both presence and action. It encompasses a shared responsibility for good and just healthcare provision by using *and transforming* all available methods. It aims to (trans-)form healthcare “from within” through Christian involvement in all its areas. Global exchange among those who share this concern is an invaluable aid to support this vision.

This White Paper is an invitation for reflection, discussion, and critical debate to explore a more inclusive form of Christian chaplaincy—one which is sensitive to differences without returning to a narrow confessionalism or an undifferentiated, neutral conception of spirituality and spiritual belonging. To meet the ongoing diversification of the spiritual–religious field, we adopt a broad and multidimensional concept of spirituality that includes equally religious and non-religious practices, experiences, beliefs, attitudes, and belonging concerning an encompassing and ultimate dimension of life for which the word “God” is used in many traditions. For chaplains, spirituality is not just something they deal with professionally in their care for patients, but a multidimensional resource from which they live and practise.

## Double Role: Witnesses and Specialists

### State of Discussion

In the countries in which the contributors of this paper are working, the status of *Christian* healthcare chaplaincy is contested from two sides. On the one hand, there are questions concerning religious/spiritual diversity, social justice, and (post-)secularity: Can healthcare chaplains embody a specific tradition without alienating those of other traditions? On the other hand, the emphasis on professionalism, scientific evidence, and accountability raises questions about the professional standards and quality management of healthcare chaplaincy. As an answer to these challenges, chaplaincy is framed increasingly as a specialized healthcare profession. Both contestations have to do with the tension between the double role of chaplains: On the one hand, as citizens and professionals, they are shaped by and rooted in a particular spiritual tradition and faith community; on the other hand, they are shaped by and committed to the secular healthcare systems in their countries. How can this tension be borne out fruitfully in today’s healthcare system(s) and pluralistic societies?

### Theological Analysis and Clinical Considerations

1. Two different narratives are available to assess the current changes in healthcare chaplaincy: The first describes this change as a transition from denominational pastoral care to a generic spiritual care, equally available for all. The second narrative sees it as another stage in the professionalization process that began as early as 1925 with Clinical Pastoral Education (CPE), a process which is not necessarily linked to de-traditionalization. While the first narrative assumes the substitution of Christian spiritual care for a secular one, the second reckons with the possibility that chaplains are both: specialized professionals in healthcare *and* witnesses or representatives of particular and highly complex traditions and communities.
2. In further professionalizing healthcare chaplaincy, both meanings of “profession” are likely to remain important: belonging to “a special kind of occupation” and “making a promise/vow” (Freidson, 1988, p. xvii). Christian chaplaincy is constituted from a double responsibility: It is (1) a response to the Christian call to healing and (2) a professional answer to the spiritual and psychosocial needs of patients and relatives. The promise that the Christian healthcare chaplain stands for is that of Christ’s healing presence and a hope beyond hope (Rom 4:18). Hence, what Christian healthcare chaplains contribute to secular healthcare does not emerge merely from themselves. As vulnerable and imperfect individuals, they are earthen vessels bearing a priceless treasure (2 Cor 4:7).
3. Christian healthcare chaplains share with their colleagues from other faith backgrounds the requirement to differentiate their professional role in a transparent manner as well as a commitment to ethical standards and specific spiritual care competencies (see Sect. "Medical Goals and the “Christian Call to Health and Healing”"). What distinguishes Christian healthcare chaplains is their theological training, their spiritual formation and practice, their rootedness in a particular spiritual tradition, and their multilayered (and at times strained) religious belonging: to Christ, to a Christian denomination, to a local church, etc. Inherent in these features of distinction is a critical potential directed against dehumanizing, exclusionary, ideological and reductionist tendencies in healthcare systems, and secular or religious communities (including those of the chaplains themselves). The critical benchmark for the work of healthcare chaplains is the best possible professional spiritual support of patients and relatives that protects and upholds their autonomy and integrity. Notably, though Christian healthcare chaplains are rooted in their tradition and communities, they, together with healthcare chaplains of other traditions, honour the particular religious and/or secular identities and communities of those to whom they provide spiritual care. No form of healthcare chaplaincy, Christian or otherwise, should be used as a tool for religious or spiritual coercion.
4. Being rooted in a spiritual community makes a difference to spiritual care by affecting its goals, quality, integrity, and authenticity. The privatization of one’s own spiritual roots strains and can even fracture the integrity and authenticity of chaplains. Spiritual care is sourced in spiritual communities; it relies on shared visions, practices, and experiences. It is the particularities of specific traditions and communities (their beliefs, languages, texts, practices, visions, etc.) that shapes and nourishes chaplains as individuals and as inter- and trans-religious teams. The care for its spiritual roots is an essential precondition of spiritual care and does not contradict the demand for further professionalization. One can work from a strong sense of belonging and yet not have to formulate it constantly. The roots do not always have to be visible, but they are there and need to be nurtured.
5. By connecting secular healthcare with spiritual traditions and communities, chaplains function as culture brokers—as intermediaries who mediate between patients, relatives, healthcare professionals, and religious communities. As mediators, chaplains occupy multiple liminalities. This puts them at risk of being marginalized, of being placed at the edge of both faith communities and medical discourses and institutions.
6. The ongoing professionalization of healthcare chaplaincy does not inevitably lead to an alienation from faith communities. However, to prevent drifting apart, a lively exchange is required by both faith communities and the chaplains themselves. From a theological perspective, healthcare chaplaincy must be viewed within the broader horizon of the larger community’s influence upon and interaction with healthcare. Healthcare chaplains hence work in frontier areas that are sources for the renewal of both their spiritual communities and of healthcare.
7. Various spiritual traditions can and should encourage one another in a common vocation of spiritual care practice. In mutual complementation and strengthening, healthcare chaplains from different traditions offer an enhancement of or corrective to medical contexts, which often attempt to focus solely on the physical (or in psychiatry: the mental). By compassionate presence, they serve as intermediaries of trust and hope, as bearers of *shalom* in the comprehensive biblical sense, and as representatives of a dimension of life called spiritual, transcendent, or otherwise.

### Perspectives and Recommendations

1. Healthcare chaplaincy can and should proactively engage the changes in society and healthcare as an opportunity to renew and advance its practice as specialists in spiritual care that belong to a particular faith tradition. Rooted in a spiritual tradition and embodying it, they engage with the spiritual beliefs and needs of patients, relatives, and professionals, and they do so within the context of a health institution and in cooperation with health professionals, other chaplains and representatives of faith/non-religious communities.
2. Healthcare chaplains need to be spiritual actors firmly rooted within a specifictradition and well accustomed to specific clinical settings (e.g. paediatrics, palliative care, psychiatry, etc.) and their language and customs. They should acquire specific professional skills to provide spiritual care respectfully to patients/relatives with different (spiritual and cultural) backgrounds—just as Jesus had to learn to care for people from other faith communities and cultures (s. Mark 5:1–20, 7:24–30; Luke 17,11–19).
3. To prevent an assimilation to secular healthcare and to safeguard the representational aspect of chaplaincy, emphasis should not be put only on formal endorsement (which is very diverse due to national and denominational differences), but also on the ways chaplains draw on the resources of spiritual traditions and communities. To counter the risk of alienation, faith communities need to proactively engage in the ongoing professionalization of healthcare chaplaincy, especially in education and training (see Sect. "Interprofessionality: Chaplaincy as Specialist Spiritual Care").
4. A formal endorsement by a faith community or indirectly by an intermediary organization can help to tie healthcare chaplaincy back to the local congregations and promote interaction between the clinical and non-clinical spheres. It situates healthcare chaplaincy within the multifaceted intersections of faith communities and the healthcare system.

## Medical Goals and the “Christian Call to Health and Healing”

### State of Discussion

The “Christian call for health and healing” (Luke 9:1–2) has been at the basis of the Christian engagement in healthcare for centuries, and it has also inspired the founders of Clinical Pastoral Education (CPE, Myers-Shirk, 2009, p. 33). What does the ecumenical reflection on the ongoing Christian “call to health and healing” (World Council of Churches, 2005, 2008, pp. 91–112) mean for the task of today’s healthcare chaplaincy? First, it raises the question about a theological understanding of distinct models of health as well as of providing a critical lens through which to consider complex interfaces within health, such as between (1) physical and mental health; (2) curing, healing and recovery; (3) disease and (mental/emotional or spiritual) distress; and (4) various forms of suffering and pain. A theological reflection on these interfaces should consider the current state of knowledge in health sciences and discuss it critically in the horizon of Christian spirituality. The clarification of the relationship between medical goals and what is intended by the Christian call for health and healing is no less urgent. Furthermore, such theological reflection is required to define the goals and scope of Christian healthcare chaplaincy. Which expectations of the health system can it fulfil and which not? What efforts need to be made to ensure that healthcare chaplains contribute optimally to palliative, rehabilitative, and curative tasks as well as to spiritual support? And how can this impact be assessed?

### Theological Analysis and Clinical Considerations

1. As mentioned in the introduction, the New Testament term *therapeuein* has a considerably broader connotation than the contemporary term *therapy*. The biblical term refers to the whole spectrum of curative medicine, spiritual healing (in different forms, cf. World Council of Churches, 2005), and care for the sick, and also encompasses social inclusion. Therefore, the Christian call for “healing” encompasses all that the parable of the Good Samaritan (Luke 10:30–35) evokes: compassionate psychosocial support, medical care and nursing, alongside healthcare’s organizational and economic or political aspects, as well as all forms of care that take place outside of healthcare institutions.
2. For a theological understanding of health and healing, the distinction between protological and eschatological aspects is crucial. From a protological perspective, God has created an abundance of remedies and gifted humankind with the creativity to invent and improve therapeutic procedures of all kinds. Therefore, God also heals by means of modern medicine, psychology, physiotherapy, etc. From an eschatological perspective, healing refers to the “reality of abundant life that breaks in through the event of Jesus Christ, the wounded healer, who participates in all aspects of human suffering, dying, and living, and overcomes violation, suffering, and death by his resurrection” (World Council of Churches, 2005, 2008, pp. 91–112). The intentionality of healthcare chaplaincy relies equally on both perspectives. To alleviate suffering and support people in critical life situations, healthcare chaplains are equipped and apply the spectrum of the (medical, psychological, and theological) knowledge and skills currently available and defined within the scope of their practice grounded in spiritual life.
3. Reducing suffering and supporting recovering and healing is not and never has been an exclusively medical task. It is, in any case, not a task that medicine can perform alone. Physicians are and remain dependent on other professions—including that of chaplaincy. Chaplaincy can contribute to a better healthcare by expanding, deepening, and complementing medical practice.
4. If chaplains see themselves in the horizon of the Christian call to healing, they can articulate their contribution to healthcare and healing without submitting to external goals that run contrary to a Christian vision for healing. Healthcare chaplaincy contributes to healthcare by its spiritual approaches, which are multifaceted in form due to different contexts and spiritual traditions. From a Christian perspective, healing concerns the interrelationship of oneself to God, others, and self. Thus, a central goal of Christian spiritual care is to support a person in finding *shalom* in the relationship with oneself, with others, and with God–amid fragmentation brought about and/or revealed by ill-health, mental distress, dying, and grief. The communication of healing presence occurs through symbolic practices of presence, which may be described as “sacramental” in a broad sense.
5. The spiritual and mental dimension of health are two distinct dimensions (World Health Organization, 2005) that are, however, intertwined. Spiritual reconnecting can decrease in mental distress and improve mental health, but it may also occur amid persistent mental suffering (cf. 2 Cor 12:7–10). On the other hand, spiritual beliefs or practices may also negatively affect mental health. This highlights the importance of professional support that is aware of the complex relationship between mental health and spirituality.
6. As “wounded healers”, healthcare chaplains transmit what they receive within their spiritual care practice. Guided by compassion, they are affected and sometimes transformed by those for whom they care.
7. Chaplaincy contributes to healthcare not least through an awareness of medical and secular reductionism, by the critique of despiritualizing systems and instrumental medical rationalities on the one hand and of a spiritual reductionism on the other hand.
8. The Christian call to healing involves both communities and professionals. Not only chaplains, but also nurses, physicians, clinical social workers, psychotherapists, and other health professionals can understand their work as an answer to the Christian call for healing. Each professional group has its own talent to bear within the context of interprofessional spiritual care. Healthcare chaplaincy can and should function as a community for equipping other healthcare practitioners in providing generalist spiritual care in the healthcare setting.

### Perspectives and Recommendations

1. If the Christian call to healing counts as one of the basic tasks of the churches, there is a need for the promotion and coordination of different institutional answers to this call. Healthcare chaplaincy should be promoted as a central way of answering this call in an increasingly diverse, complex, and specialized healthcare.
2. That chaplaincy has a healing effect should be asserted and made comprehensible as far as possible. Empirical research, Patient-Reported Outcome Measures (PROM), case studies, etc., could contribute to a more profound understanding of the therapeutic aspects of healthcare chaplaincy.

## Professional Spiritual Care Competencies

### State of Discussion

Healthcare chaplaincy is based on a broad spectrum of professional competencies anchored in a spiritual aptitude. While it is easy to name the communicative competencies, the description of the spiritual dimension of these competences or specific spiritual competencies is more challenging.

### Theological Analysis and Clinical Considerations

1. Spiritual care competence encompasses: a spiritual attitude; the ability to touch upon and explore the spiritual beliefs and needs of patients and their relatives; the ability to accompany people with diverse beliefs and belongings as well as to deal professionally with misunderstandings and conflicts arising from religious–spiritual and cultural diversity (“diversity competence”); the competence of healing presence; the competence to perform spiritual rituals in a clinical context; ethical competence needed in dealing with conflict, ambivalence, cultural and religious diversity, and difficult treatment decisions; spiritual self-care and having an awareness of one’s own vulnerability and needs, etc. Not all dimensions of spiritual care competence can be taught and evaluated equally well.
2. The teachable and testable aspects of a spiritual care competence encompass theological, ethical, health and disease-related knowledge, as well as specific practical skills, like hermeneutic communication. At least partially learnable and evaluable is a spiritual and self-reflexive attitude.
3. The theoretical side of a spiritual care competence includes, among others, reliable knowledge regarding religious and spiritual diversity, pastoral care and a theological understanding of health, illness, and healing, as well as basic knowledge in human sciences. Not least, it encompasses the ability to relate theological and social scientific knowledge to each other.
4. The practical side of spiritual care competence includes three domains: First, the further development of the students’ spirituality with regard to the work of healthcare chaplaincy and cultural and religious diversity; second, the capability to assess and address the spiritual needs of patients, relatives, and professionals in a patient-centred, culturally sensitive, and resource- and process-oriented manner (including professional approaches to misunderstandings and conflicts arising from religious–spiritual and cultural diversity); and third, the ability to foster the integration of interprofessional spiritual care on an organizational and societal level. The *first* domain includes, among others, the mapping of one’s own spirituality, and the identification of pathways for further development. The *second* domain includes, among others, the following teachable and learnable skills: to perceive and assess spiritual distress and spiritual resources; to relate the patients’ (and their families, as relevant) story and present situation to his or her spiritual/worldview identity and resources; to help patients’ and their families discover and renew sources of spirituality and belief; to select, adapt, and perform rituals and prayers; to use digital tools for spiritual care, etc. The *third* domain includes, among others, the chaplain’s ability to articulate their professional identity and the goals of their work (generally and in particular cases) in a language understandable for healthcare professionals and to document spiritual care concisely in patient records.

### Perspectives and Recommendations

1. Spiritual care competence, like any other professional competence, is something that must be worked on throughout a career, starting from basic training to continuing education. Churches and professional associations should pursue an active policy to support chaplains in this regard. This involves facilitating training, mutual communication, and evaluating development (for example, by means of an accreditation system). For universities, colleges, and other spiritual caregiver training programmes, the challenge is to develop the training in spiritual care competence in consultation with diverse faith communities and sending bodies.
2. With regard to interprofessional spiritual care (see next section), spiritual care competence must be specified relating to different professional realms (chaplaincy, medicine, nursing, psychotherapy, etc.) and healthcare domains (palliative care, mental health, etc.).
3. As there is no consensus on what exactly the necessary spiritual care competences are, different levels should be clarified nationally and internationally in a consensus process. An international exchange about different evaluation procedures and the experiences with them is also desirable.

## Interprofessionality: Chaplaincy as Specialist Spiritual Care

### State of Discussion

Spiritual care is not a domain of responsibility reserved only for healthcare chaplains, but uniquely shared in partnership with all healthcare professions. Understanding spiritual care as an interprofessional task raises the question of professional responsibilities, competencies, and the specific role of healthcare chaplains. To clarify different roles and responsibilities, the distinction between *generalist* (not “generic”) and *specialist spiritual care* has become internationally used (Puchalski et al., 2014). Healthcare chaplains are specialists in a field where nurses, doctors, and allied health professionals are primary caregivers. How can this distinction be understood and endorsed theologically, and what does it mean for the development of Christian healthcare chaplaincy?

### Theological Analysis and Clinical Considerations

1. The Christian call to heal was addressed to “care teams” from the beginning, as Jesus did not send his disciples out alone, but in pairs (Mark 6:7; Matthew 10:5–6; Luke 9:1–6). The Christian tradition has a long and tense history of professionalization, and of multi-, inter-, and transprofessional collaboration. The Emmanuel Movement in Boston, the St Christopher’s Hospice in London, the Christian Medical College in Vellore, India, and the Christian Medical Commission in Geneva stand, among others, for fruitful syntheses of this tradition and modern medicine.
2. Interprofessionality in spiritual care is the best way to address the spiritual needs of patients and relatives in highly specialized healthcare institutions. Interprofessional cooperation ensures a continuity of spiritual care.
3. To have a clear role among healthcare professionals, it is in chaplains’ best interest to define their work within the frame of healthcare, where it is common to distinguish between different degrees of specialization. The distinction between generalist and specialist spiritual care both clarifies particular roles and underlines the need for increased synergy and partnership. It helps to keep in view what is distinctive about the complementary professional approaches, while at the same time relating them to each other. In a specialist environment, chaplains are generalists in psychosocial support and specialists in the spiritual dimension. Ideally, physicians, nurses, psychotherapists, and social workers are—each in a profession-specific way—generalists in spiritual care.
4. From the healthcare perspective, this distinction is about professional specialization; from the theological perspective, it is also about the representational aspect of chaplaincy. Chaplains stand for a particular faith tradition in secular health institutions. At the same time, they are contact points for religious and spiritual concerns in general. They are distinguished *ex professo* by the fact that their own situation in the field of religion and spirituality is, to a certain extent, connected to their professional role and thus must be declared if asked.

### Perspectives and Recommendations

1. To ensure that the spiritual needs of patients and relatives do not get lost in highly specialized healthcare institutions, spiritual care should be implemented as a common interprofessional task with clarified roles, responsibilities, and procedures.
2. Both generalist and specialist spiritual care need corresponding competencies (see previous section) and thus training and ongoing supervision and continuing education.
3. Healthcare chaplains who retreat to an island where they can continue to speak their own language are an obstacle to fruitful cooperation between medical, psychological, social, and spiritual care. Thus, chaplains should develop and use a common language for spiritual needs, resources, and support that non-theologians can understand and use. While biblical texts and religious language are the main resource for chaplains themselves, and possibly for patients who belong to the same tradition, chaplains also need to serve as translators between separate languages of health and healing, offering an interpretation that helps bridge the divide between faith and the human sciences and between different faith traditions.
4. Organizing spiritual care calls for partnership and cooperation. Documenting spiritual care is essential for an interprofessional approach in highly specialized institutions.
5. To ensure continuity of spiritual care, new models of networking between inpatient and outpatient support are needed. Telechaplaincy and specialist spiritual care teams for outpatient care offer promising pathways for this. Healthcare chaplains can function as gatekeepers and networkers.

## Education and Training of Healthcare Chaplains

### State of Discussion

Healthcare chaplaincy has relied heavily on CPE as an essential formative programme in parts of the world. As such, CPE has relied on theological education to prepare students for clinical settings. As CPE is not a programme in other parts of the world, there never was one coherent, worldwide training of healthcare chaplains. On the contrary, chaplaincy training programmes exist in a wide variety of durations and contents all over the globe. But even in countries where CPE was the leading programme, new models are emerging. Critical enquiries are coming from two directions: On the one hand, there is the question of how CPE is prepared for the social developments mentioned in the preceding sections. On the other hand, outcome-oriented chaplaincy has questioned the CPE’s reliance on a model of counselling developed in the 1940s by Carl Rogers. Is healthcare chaplaincy on the verge of an opportunity to reconceptualize CPE, turning from its Christian (and primarily Protestant) roots to a more multi-faith/multi-meaning-making frame? How is it possible to form Christian chaplains for secular and/or pluralist contexts in ways that have both substance and integrity? Does the emphasis on “outcome” in current healthcare chaplaincy research (and other factors, e.g. digitalization, pluralization, and secularization) lead to a (paradigm) shift within CPE? To what extent is a “presence orientation” compatible with a stronger reflection on professional objectives in the field of spiritual care?

### Theological Analysis and Clinical Considerations

1. The understanding of healthcare chaplaincy as an autonomous profession requires a suitable curriculum for a highly complex and (in terms of spiritual beliefs, practices, and belonging) increasingly pluralistic professional field. The more the training of chaplains is detached from the study of theology, the more the question arises: How can the requisite theological knowledge be integrated into chaplaincy training?
2. An education for healthcare chaplains focused on the aforementioned spiritual care competencies can build on the foundations of CPE, especially in terms of enabling individualized learning processes. At the same time, such an approach means a stronger focus on assessable learning content and objectives.
3. The education and training of healthcare chaplains encompass informative, formative, and transformative learning (Frenk et al., 2010). Through *informative* learning, chaplaincy students acquire reliable theological knowledge (including an understanding of diverse faith traditions and worldviews) as well as basic knowledge in health sciences (see 3.2.3). By means of *formative* learning, practical spiritual care competencies are acquired (see 3.2.4–3.2.6). *Transformative* learning fosters the ability for a compassionate presence as well as spiritual integration and development. Furthermore, transformative learning involves the development of innovative spiritual leadership in a time of change and of increasing diversity.
4. Outcome-oriented chaplaincy emphasizes clarifying chaplaincy objectives, documentation, and empirical chaplaincy research. However, such an outcomes-oriented approach has raised tensions with the CPE training model since its inception, given CPE’s emphasis on presence and the shedding of specific agendas. Healthcare chaplaincy should pursue efforts to quantify the effectiveness of chaplaincy work and to review it continually within an appropriate quality management framework, together with refining extant non-outcomes-oriented approaches to chaplaincy.
5. There is more commonality than difference in the training of healthcare chaplains with different religious and spiritual backgrounds. Hence, joint training can be an opportunity for all trainees to gain better understandings of other spiritual and cultural traditions (Szilagyi et al., 2024). A precondition for such learning is an approach that values and strengthens chaplaincy students’ disparate religious and spiritual identities.

### Perspectives and Recommendations

1. To address the needs of a professional domain in which scientific evidence is highly valued, and to harness and foster the research on spiritual care, healthcare chaplaincy should remain a profession with an anchoring in academia. Integrating volunteers in healthcare chaplaincy may be helpful, but they need professional education and training in spiritual care and ongoing supervision to avoid the risk of de-professionalization. Interdisciplinary research and scholarship, together with literacy in different disciplines, is urgently needed, combining empirical and theological approaches to healthcare chaplaincy.
2. Healthcare chaplains need to be (a) spiritual actors based within a specific faith tradition, (b) hermeneutically competent and well-informed in theology and religious sciences, (c) well accustomed to specific clinical settings, their language, and customs, (d) have specific professional skills to provide spiritual care to individuals with different (spiritual and cultural) backgrounds, (e) be able to articulate and justify their goals and procedures in medical and religious contexts, and (f) be aware of their own spiritual resources as well as of their own vulnerability and needs.
3. If the training of healthcare chaplains shifts away from a theological master’s degree as a precondition, requisite theological training must be redefined together with alternative modes of spiritual formation. How such training is integrated as part of new curricula, where and how they are delivered, and by whom, are key attendant questions to be addressed.
4. The forms of learning must be oriented in such a way that they enable informative, formative, and transformative learning processes in a balanced measure. These include among others: blended learning and flipped classroom approaches, analysing case studies and verbatims, supervision, internships, learning with and from patients, and spiritual accompaniment (individually and in groups). To integrate theological and empirical perspectives, interdisciplinary team teaching and interprofessional team learning is highly recommended (World Health Organization, 2010). As modular curricula are the rule now, they should encompass interfaith and faith-specific modules in a balanced way.
5. An outcome orientation must take seriously the empirical evidence that the effectiveness of chaplains lies particularly in relational competence, and their ability to offer a caring and compassionate presence. Moreover, to be effective and accountable, chaplains should learn to formulate their goals clearly (which also include a non-directive approach) and create space for the paradox of intentional non-intentionality to unfold. This is based on trust in a healing and transformative presence, which is revered in Christianity as the Holy Spirit.

## Conclusion

The changes in society and healthcare described in this White Paper profoundly challenge healthcare chaplaincy. They urge chaplains with different spiritual backgrounds to clarify their professional self-understanding and embedding in interdisciplinary care teams, their understanding of health and healing, their approach to religious–spiritual diversity, and the education and training needed to fulfil their tasks. In facing these challenges, the authors of this ecumenical paper share the following convictions:

- The Christian call to healing, to which healthcare chaplaincy can also contribute, requires new answers under changed conditions;
- An interprofessional approach is the best way to address spiritual needs in highly specialized healthcare institutions, and healthcare chaplains are specialists in this field;
- The education and training of healthcare chaplains encompasses informative, formative, and transformative learning;
- To become a *Christian* healthcare chaplain, this learning process has a specific focus on Christian theology of health and healing and is firmly rooted in Christian spirituality and congregational life.

This White Paper aims to stimulate critical discussion among all those involved in healthcare chaplaincy. It proposes that healthcare chaplains should be recognized as specialized professionals in healthcare *and* as witnesses of a particular spiritual tradition and of the spiritual dimension of life in general. Believing that God’s spirit can strengthen, comfort, and heal people in various ways—through the means of modern medicine, care, psychology, and counselling, through prayer and spiritual practice of all kinds, as well as through direct presence and unforeseeable experiences—Christian healthcare chaplains respond professionally to the spiritual and psychosocial needs of those whom they serve, and to the development of interprofessional spiritual care in late-modern healthcare. In close collaboration with healthcare professionals and chaplains of other traditions, they honour and serve patients, relatives, and medical staff without discrimination, thus valuing their dignity and personhood. In this way, healthcare chaplains contribute to a better healthcare by expanding, deepening, and completing medical practice.

## Footnotes

1 The term “Christian healthcare chaplains” herein refers to professional chaplains who understand their role in terms of the Christian gospel. This includes both professionals who are employed and publicly identifiable *as Christian* (and having a particular denominational background), as well as chaplains with a Christian background employed in contexts where religious affiliation is not made explicit in the context of care. When we speak of “healthcare chaplaincy” or “spiritual care”, we have primarily its Christian forms in mind (in various healthcare institution: hospitals, psychiatric and psychosomatic clinics, hospices, etc.), though we are convinced that much of it applies to non-Christian chaplains as well.
