# Correction to: Spiritual Care at the Crossroads: An Ecumenical White Paper on the Future of Christian Healthcare Chaplaincy

**DOI:** 10.1007/s10943-025-02397-1

**Published:** 2025-08-04

**Authors:** Simon Peng-Keller, Michael Balboni, Tracy Balboni, Annette Haussmann, Trace Haythorn, Pascal Mösli, David Neuhold, Daniel R. Nuzum, Wim Smeets, Chris Swift, John Swinton, Traugott Roser, Anne Vandenhoeck, Fabian Winiger

**Affiliations:** 1 Faculty of Theology and the Study of Religion, University of Zurich, Kirchgasse 9, 8001 Zurich, Switzerland; 2 Harvard Medical School Boston, Boston, MA USA; 3 Faculty of Theology, Heidelberg University, Heidelberg, Germany; 4 Chaplaincy Innovation Lab, Decatur, GA USA; 5 Faculty of Theology, University of Lucerne, Lucerne, Switzerland; 6 Department of Obstetrics & Gynaecology, University College Cork College of Medicine and Health, Cork, Ireland; 7 Clinical Pastoral Education, Cork University Hospitals Group, Cork, Ireland; 8 Radboud University, Nijmegen, NL The Netherlands; 9 Stafordshire University, York, UK; 10 School of Divinity, History and Philosophy, King’s College, University of Aberdeen, Aberdeen, UK; 11 Faculty of Protestant Theology, University of Münster, Münster, Germany; 12 Faculty of Theology and Religious Studies, KU Leuven, Louvain, Belgium

**Correction to: Journal of Religion and Health (2025) 64:1031–1046** 10.1007/s10943-025-02255-0

In this article, the affiliation details for Author Fabian Winiger were incorrectly given as “Faculty of Theology, University of Lucerne, Lucerne, Switzerland” but should have been ‘Faculty of Theology and the Study of Religion, University of Zurich, Kirchgasse 9, 8001 Zurich, Switzerland’.

The original article has been corrected.

